# Functional Genomics Uncovers Pleiotropic Role of Rhomboids in *Corynebacterium glutamicum*

**DOI:** 10.3389/fmicb.2022.771968

**Published:** 2022-02-21

**Authors:** Andrea Luenenschloss, Frank ter Veld, Stefan P. Albaum, Tobias M. Neddermann, Volker F. Wendisch, Ansgar Poetsch

**Affiliations:** ^1^Plant Biochemistry, Ruhr University Bochum, Bochum, Germany; ^2^Center for Biotechnology, Bielefeld University, Bielefeld, Germany; ^3^Faculty of Biology, Bielefeld University, Bielefeld, Germany; ^4^Department of Marine Biology, Ocean University of China, Qingdao, China; ^5^Qingdao National Laboratory for Marine Science and Technology, Qingdao, China

**Keywords:** membrane protease, heat stress, physiological adaptation, corynebacteria, proteomics

## Abstract

The physiological role of ubiquitous rhomboid proteases, membrane-integral proteins that cleave their substrates inside the lipid bilayer, is still ill-defined in many prokaryotes. The two rhomboid genes *cg0049* and *cg2767* of *Corynebacterium glutamicum* were mutated and it was the aim of this study to investigate consequences in respect to growth phenotype, stress resistance, transcriptome, proteome, and lipidome composition. Albeit increased amount of Cg2767 upon heat stress, its absence did not change the growth behavior of *C. glutamicum* during exponential and stationary phase. Quantitative shotgun mass spectrometry was used to compare the rhomboid mutant with wild type strain and revealed that proteins covering diverse cellular functions were differentially abundant with more proteins affected in the stationary than in the exponential growth phase. An observation common to both growth phases was a decrease in ribosomal subunits and RNA polymerase, differences in iron uptake proteins, and abundance changes in lipid and mycolic acid biosynthesis enzymes that suggested a functional link of rhomboids to cell envelope lipid biosynthesis. The latter was substantiated by shotgun lipidomics in the stationary growth phase, where in a strain-dependent manner phosphatidylglycerol, phosphatidic acid, diacylglycerol and phosphatidylinositol increased irrespective of cultivation temperature.

## Introduction

In their natural habitat, bacteria must constantly adapt to changing environmental conditions, such as a sudden increase in temperature. Consequently, organisms have evolved transcriptional, translational, and post-translational regulation mechanisms for adapting their physiology. One strategy is the use of regulated intramembrane proteolysis, accomplished by proteases with their cellular membrane-embedded catalytic center (Urban and Freeman, 2002). Rhomboids comprise the largest family of all the intramembrane proteases (Weihofen and Martoglio, 2003; Urban and Dickey, 2011); genome sequence analysis revealed their almost ubiquitous occurrence in archaea, bacteria, and eukaryotes, though protein sequence homologies can be as low as 6% (Lemberg and Freeman, 2007). The first evidence for proteolytic function of rhomboids was obtained for rhomboid-1 in *Drosophila melanogaster*, which is responsible for liberation of the epidermal growth factor (EGF) from Golgi membranes (Lee et al., 2001).

Rhomboids are a rare case of membrane proteins, where structure and mechanism have been explored at an advanced level, whereas our knowledge on physiological function and natural substrates is falling short for most bacteria. For several years, only one validated substrate for a bacterial rhomboid, TatA of *Providencia stuartii* has been unveiled (Stevenson et al., 2007). Cleavage of its N-terminal domain by the rhomboid AarA activates TatA for quorum signal liberation. Unfortunately, this N-terminal domain is absent in the vast majority of bacterial A subunits of the Tat exporter, hence TatA processing does not provide a hallmark on prokaryotic rhomboid substrates and function. In *Bacillus subtilis*, deletion of the rhomboid-encoding *gluP (yqpP)* gene lead to an incomplete, filamentous cell division and slightly impaired glucose import (Mesak et al., 2004). Moreover, function of GluP as adaptor and protease for magnesium transporter MgtE was found (Began et al., 2020). Ablation of GlpG does not produce any morphological changes, but renders *Escherichia coli* more resistant against the β-lactam antibiotic Cefotaxime (Clemmer et al., 2006). Lately, for *Shigella sonnei* involvement of rhomboids GlpG and Rhom7 in membrane protein quality control with HybA, FdnH, and FdoH as substrates was demonstrated (Liu et al., 2020).

Whereas bacteria usually possess one rhomboid gene, *Corynebacterium glutamicum* possesses two, namely *cg0049/NCgl0043* and *cg2767/NCgl2426*, both with unknown function. *C. glutamicum* is a mycolic acid-containing actinomycete, Gram-positive, GC-rich, non-sporulating, immotile soil bacterium and has been used extensively in industrial biotechnology, mainly for fermentative production of the amino acids L-glutamate and L-lysine (Wendisch, 2020). Despite its close relationship to pathogenic bacteria like *Mycobacterium tuberculosis* and *Corynebacterium diphtheriae, C. glutamicum* is generally recognized as harmless for humans and has a history of more than 50 years of safe use in food biotechnology. Direct substrates of rhomboids have not been described in its pathogenic relatives.

Given the limited knowledge about the physiological role of rhomboids in prokaryotes, the aim of this study was to illuminate rhomboid function by employing a functional genomics approach that combines shotgun proteomics and DNA microarray analysis for *C. glutamicum* strains. For this purpose, a double deletion strain devoid of *cg0049* and *cg2767* was constructed and together with the wild type subjected to comparative – omics analysis under various growth conditions, which revealed that rhomboids are involved in a broad range of cellular processes.

## Materials and Methods

### Experimental Design

*Corynebacterium glutamicum* was cultivated at 30 or 40°C and cells harvested in the exponential and stationary phase. Three biological replicates were used for proteomics and lipidomics, two for transcriptomics. Differences between samples were determined using Student’s *t*-test and ANOVA for proteomics.

### Construction of the Rhomboid Double Deletion Mutant

Used strains, plasmids, and oligonucleotides are listed in Table 1. SOE-PCR (Horton et al., 1990) with *cg2767* upstream and downstream primer pairs yielded a 3′-truncated *cg2767* gene sequence. This approach resulted in a 5′-truncated *cg2767* gene for the *C. glutamicum*Δ*cg2767* strain to retain nucleotide sequences in the chromosome that overlap with predicted regulatory sites of the downstream *marR* gene. The PCR product was cloned in the pK18*mobsacB* vector for replication in *E. coli*. Allelic exchange of the *cg2767* gene in *C. glutamicum* ATCC13032 was obtained by transformation with the pK18*mobsacB*-del-*cg2767* (Table 1) and selection for double crossover events as described (Schafer et al., 1994). Likewise, in *C. glutamicum* ATCC13032 Δ*cg2767* additional deletion of *cg0049* was achieved with pK18*mobsacB*-del-*cg0049* to construct the final mutant *C. glutamicum* ATCC13032 Δ*cg2767*/Δ*cg0049*. Chromosomal deletion/truncation of *cg0049* and *cg2767* was confirmed by PCR with primers complementary to gene flanking regions (data not shown).

**TABLE 1:** Used strains, plasmids, and oligonucleotides.

*C. glutamicum* ATCC13032	Wild type strain	Abe et al., 1967
*C. glutamicum* ATCC13032 Δ*cg2767*	Δ*cg2767*	This study
*C. glutamicum* ATCC13032 Δ*cg2767* Δ*cg0049*	Δ*cg2767*,Δ*cg0049*	This study
*E. coli* DH5α	U169*deoRrecA1endA1hsdR17 (rK-mK*+) *sup44thi-1 gyrA69*	Hanahan, 1983
pK18*mobsacB*	Vector for allelic exchange in *C. glutamicum*, pK18 *oriV*_E.c._, Km^r^, *sac*B	Schafer et al., 1994
pK18*mobsacB*-del-cg*2767*	Vector with insert of PCR-amplified *cg2767* upstream and downstream regions	This study
pK18*mobsacB*-del-*cg0049*	Vector with insert of PCR-amplified *cg0049* upstream and downstream regions	This study
cg0049 upstream 1	GCGGATCCGTCTCTACAGCCTGCTCAGCTTTTGGTGC	For *cg0049* deletion, *Bam*HI restriction site
cg0049 upstream 2	GAGGGCAATGGGCGGAATGTGCAGGTTTTTGTGGCAT	Base pairing with cg0049 downstream 1
cg0049 downstream 1	ATGCCACAAAAACCTGCACATTCCGCCCATTGCCCTC	Base pairing with cg0049 upstream 2
cg0049 downstream 2	GCGGTCGACTCTTTGGCAGTTGATCTTGC	*Sal*I restriction site
cg2767 upstream 1	GCGGATCCCCAAGCAGTGCAACGGAAACTCC	For *cg2767* deletion, *Bam*HI restriction site
cg2767 upstream 2	GCTTCAGGGAATTTTACCGATTTTCGGTGGAATCGGCA	Base pairing with cg0049 downstream 1
cg2767 downstream 1	GTGTAGTCGCTAGGTGAAAAGGTAAAAGTTCCCTGAAGC	Base pairing with cg0049 upstream 2
cg2767 downstream 2	GCGGTCGACACGGGTTGCTCAGTGGTCATTGC	*Sal*I restriction site

*Underlined terms indicate BamHI restriction site.*

### Cultivation of *C. glutamicum* and Heat Shock Experiments

*Corynebacterium glutamicum* ATCC 13032 and *C. glutamicum* ATCC 13032 *Δcg2767*/*Δcg0049* cells were cultivated at 30°C on a rotary shaker at 150 rpm. For proteome and transcriptome analyses, a first preculture was grown in 10 ml BHI medium (Becton, Dickinson and Company, Sparks, MD, United States), followed by an overnight cultivation in 50 ml minimal medium I (Kase and Nakayama, 1972). Main cultures were inoculated with the overnight cultures in 100 ml minimal medium. For proteome and transcriptome analyses in the exponential growth phase cells were cultivated to an OD600 ∼ 0.7 and then shifted to 40°C for 2.5 h (proteome) or 2 h (transcriptome). For proteome survey in the stationary phase wild type and mutant strain were grown to OD600 ∼ 30 and subsequently exposed to heat treatment of 40°C for 2.5 h.

For the isotopically labeled internal standard *C. glutamicum* ATCC 13032 was cultivated in modified MMES medium (Koch et al., 2005), containing ^15^NH_4_Cl as nitrogen source. Heat shock was performed for 2.5 h either at OD600 ∼ 0.7 or OD600 ∼ 30.

For Western blot detection of Cg2767, cells in the stationary phase were shifted for 2.5 h from 30 to 40°C in the stationary growth phase and from 30 to 50°C in the exponential growth phase.

### DNA Microarray Analysis

Fifteen milliliters *C. glutamicum* cells in the exponential growth phase were pelleted by centrifugation. For cell breakage and lysis the pellet was resuspended in 400 μl lysis buffer of RNeasy Mini Kit (Qiagen, Hilden, Germany) supplemented with 1% (v/v) β-mercaptoethanol and transferred into cryo-vials containing 300 mg glass beads (400–600 μm diameter, Sigma-Aldrich). After vial insertion, the Precellys homogenizer (Bertin Technologies, Bordeaux, France) was set to 6,800 rpm for 30 s, 30 s break, three cycles. All subsequent procedures were done according to the instructions of the manufacturer, and RNA was eluted from the columns with deionized water. The generation of whole-genome DNA microarrays (Wendisch, 2003), synthesis of fluorescently labeled complementary DNA from total RNA, microarray hybridization, washing, raw data analysis, and statistical analysis were performed as described (Ishige et al., 2003; Lange et al., 2003; Polen and Wendisch, 2004). Genes were considered as differentially expressed, if the following criteria were fulfilled: at least twofold changes in expression, adjusted *p*-value of *t*-test < = 0.05, spot signal intensity (*a*-value > 8).

### Antibody Generation and Detection of Cg2767

A rabbit polyclonal antibody was generated against the C-terminal amino acid sequence LKAKKQQKKLEKQQRQRGL of Cg2767. Peptide synthesis, cross-linking to KLH carrier protein, rabbit immunization, and collection of sera was ordered from Microsynth Seqlab GmbH, Göttingen, Germany. Antibody cross-reactivity required affinity further purification with the peptide coupled to epoxy-activated sepharose 6B beads (GE Healthcare, Munich, Germany). Peptide coupling and affinity chromatography was essentially carried out as described by the chromatography material supplier. Antibody was eluted with 0.1 M glycine, pH 2.5 and immediately mixed with the 1 M Tris/HCl pH 8.5 storage buffer. For the detection of Cg2767, proteins were separated on SDS gels (*T* = 12.5%, *C* = 2.6%) (Laemmli, 1970) and blotted on PVDF membranes (Pluskal et al., 1986) (Roche Diagnostics GmbH, Mannheim, Germany) in a semidry transfer apparatus with a buffer containing 25 mM Tris, 192 mM glycine, 0.1% (w/v) SDS, pH 7.8. The membranes were labeled with primary antibody 1:3500 diluted in PBS/Tween (8 g/L NaCl, 0.2 g/L KH_2_PO_4_, 1.4 g/L Na_2_HPO_4_, 0.2 g/L KCl, 0.5% w/v Tween 20) and 1:10000 diluted peroxidase-conjugated goat anti-rabbit immunoglobulin (Sigma-Aldrich, Steinheim, Germany). Proteins were visualized by chemiluminescence using Immobilon™ Western chemiluminescent HRP Substrates (Millipore Corporation, Billerica, MA, United States) according to the instruction of the manufacturer.

### Subcellular Fractionation and Protein Preparation

Cell disruption and membrane preparation were performed according to Haussmann et al. (2009). In addition to the membrane proteins, after ultracentrifugation soluble cytosolic proteins from the supernatant were analyzed.

For preparation of secretory proteins cells were harvested twice at 4,500 × *g* for 15 min (4°C). Supernatant was collected and 0.1% TCA (pH < 4) was added. Fifteen milliliters of the supernatant were concentrated using Amicon Ultra-15 Centrifugal Filter Unit with Ultracel-3 membrane (Millipore Corporation, Billerica, MA, United States). Remaining protein solution was stored at −80°C.

Protein concentrations were determined using DC Protein Assay (Bio-Rad, Hercules, CA, United States) according to company information.

Equal amounts of ^15^N-labeled internal standard for 30 and 40°C of membrane, cytosolic or secretory proteins of *C. glutamicum* ATCC 13032 were mixed and pooled with the same quantity of proteins of the wild type or mutant ^14^N samples. Samples were loaded on a SDS-PAGE (*T* = 12.5%, *C* = 2.6%) according to Laemmli (1970) and separated until they reached the separation gel. Proteins were stained by Coomassie Brilliant Blue as described by Dyballa and Metzger (2009). Protein bands were excised and completely destained according to Schluesener et al. (2005). After drying in the SpeedVac the gel pieces were subjected to tryptic protein digestion by the addition of 50 μL from a 12.5 ng/mL trypsin solution (sequencing grade, Promega, Madison, WI, United States) in 25 mM ammonium bicarbonate, pH 8.6 overnight at 37°C. The gel-eluted tryptic digest was dried in the SpeedVac and subsequently resuspended in 10 μl buffer A (0.1% formic acid in water, ULC/MS, Biosolve, France, Netherlands) by sonication for 5 min. The samples were transferred in LC-MS grade glass vials (12 mm × 32 mm glass screw neck vial, Waters Corporation, United States).

### Mass Spectrometry

For one-dimensional nLC-ESI-MS/MS analysis of the digested gel slices, a nanoAcquity UPLC system (Waters Corporation, Milford, MA, United States) was coupled to a LTQ Orbitrap Velos (Thermo Fisher Scientific, Waltham, MA, United States). The nanoAcquity UPLC system was equipped with an analytical UPLC HSS T3 column (1.8 μm, 75 μm × 250 mm nanoAcquity UPLC column, Waters Corporation, United States) and a PicoTip Emitter (Silica Tip™, 20 μm i.d., New Objective, Woburn, MA, United States). The system was operated at a flow rate of 300 nL/min. Eight microliters of the sample were loaded directly onto the analytical column using the nanoAcquity autosampler and bound with 99% buffer A (0.1% formic acid in water, ULC/MS, Biosolve, Netherlands) for 65 min. Peptides were eluted by a gradient with increasing acetonitrile concentration from 1 to 99% (buffer B: 0.1% formic acid in acetonitrile, ULC/MS, Biosolve, Netherlands), whereby the data acquisition was started with 60 min delay. For complex membrane or cytoplasmic protein fractions, a 380 min gradient was used. After 65 min at 1% acetonitrile, the concentration was increased to 3% within 20 min, followed by a linear gradient to 30% acetonitrile in 225 min (65–85 min: 3% buffer B; 85–205 min: 13% buffer B; 205–265 min: 20% buffer B; 265–310 min: 30% buffer B). To elute all peptides from the column, the acetonitrile concentration was raised to 99% in 25 min and kept constant for 5 min before the column was re-equilibrated at 1% acetonitrile within 40 min. The LTQ Orbitrap Velos was operated via instrument method files of Xcalibur (Rev. 2.1.0). The dual ion trap and the orbitrap were operated in sequence, i.e., during a full MS scan on the orbitrap in the range of 300–1,700 m/z at a resolution of 60,000. MS/MS spectra of the ten most intense precursors were detected in the ion trap. The analytical column oven was set to 45°C and the heated desolvation capillary was set to 275°C. The relative collision energy for collision-induced dissociation was set to 35%. Dynamic exclusion was enabled with a repeat count of one and a 45 s exclusion duration window. Singly charged and more than triply charged ions were rejected from MS/MS. A spray voltage of 1.3–1.8 kV was applied. For all performed analyses three biological replicates were measured.

### Protein Identification and Quantification

Protein identification using a *C. glutamicum* database (version 08042010) containing 3,058 entries (Kalinowski et al., 2003) was performed by SEQUEST algorithm embedded in Proteome Discoverer 1.3 (Thermo Fisher Scientific, Waltham, MA, United States) and the following result filter criteria were used: up to two missed tryptic cleavages, 5 ppm precursor mass accuracy, 0.8 Da fragment mass tolerance, methionine oxidation as variable modification two unique peptides per protein, and peptide false discovery rate (q-score) of 0.01. Search results were saved as text files.

Protein quantification by metabolic labeling was performed with QuPE (Albaum et al., 2011). For quantification Raw-files were converted into mzXML-files by use of MassMatrix File Conversion Tools 3.9^1^. Text-files and mzXML files were submitted to QuPE quantification. In QuPE the quantification utilizing peptide elution was performed, using the default filter. All results were normalized with the center on sample’s median. ANOVA and protein statistics were executed according to QuPE default settings, *p*-values adjusted for multiple hypotheses testing according to Bonferroni-Holm. After evaluation of the best clustering method and the cluster size, a hierarchical cluster analysis was performed. Cluster sizes were chosen as recommended, with the parameters ward as method for the linkage and Euclidean as distance method. To determine the optimal number of clusters in each dataset the cluster index of Krzanowski and Lai (1988) was employed. Aim of this cluster index is to identify the clustering which best groups similar proteins in the same cluster while the clusters among each other are utmost differently.

### Shotgun Lipidomics

Lipids were extracted from whole cells containing 3–8 mg cell material in 20 μl H_2_O with ten volumes of *n*-butanol (Bjerve et al., 1974), and the internal standard 1,1′,2,2′-tetramyristoyl cardiolipin (Avanti Polar Lipids, Inc., Alabaster, AL, United States) was added. The sample was mixed vigorously at 70°C using a dry block heating mixer at 1,200 rpm for 15 min. Subsequently, 0.3 mL of H_2_O was added and the sample was centrifuged at 1,000 × *g* for 5 min. The supernatant was collected and the organic phase was evaporated using a SpeedVac^®^ concentrator. Next, dried lipid extracts were subjected to a two-phase re-extraction with 0.8 mL mixture of chloroform/methanol/water at 1:2:4 (v/v/v) to remove further remaining impurities. Upon centrifugation at 1,000 × *g* for 5 min, 0.2 mL of organic phase aliquot was taken and stored at −20°C for subsequent analysis.

Electrospray ionisation (ESI) mass spectrometry was carried out using a Thermo LTQ Orbitrap XL mass spectrometer (San Jose, CA, United States) equipped with a heated electrospray ionization (HESI) source. The sample was introduced into the ESI source by direct infusion at a flow rate of 10 μL/min for 4 min. The mass spectrometer was operated in negative ESI mode. The signal was tuned for the transmission and detection of phosphatidylglycerol ions. Tune parameters were optimized using standards: positive spray voltage, 4.5 kV; negative spray voltage, 5.0 kV; capillary temperature, 270°C; declustering voltage, 10 V; sheath gas pressure, 5 au (arbitrary units); ion sweep gas pressure, 0 au; auxiliary gas pressure, 5 au; negative tube lens voltage, 110 V; positive tube lens voltage, 60 V; activation type, HCD; normalized HCD collision energy, 40. Xcalibur software version 2.1 (Thermo Fisher Scientific Inc., Waltham, MA, United States) was used for data acquisition and analysis.

Upon high-resolution precursor scanning at 100,000 (Full Width at Half Maximum, FWHM), MS/MS spectra were acquired at 7,500 FMHW with the Orbitrap (FT) analyzer: minimal precursor signal intensity, 200 counts; precursor isolation width, 1.8 Th; maximum injection time, 200 ms; target value for automated gain control (AGC), 200,000 ions.

Survey spectra from 500 to 1,300 *m/z* and data-dependent MS/MS spectra were obtained in a propriety “Nth Order Double Play” mode offered by Xcalibur. Target resolution in full-scan mode was 100,000 FMHW, followed by the acquisition of 10 HCD FT MS/MS daughter spectra at 7,500 FMHW of the most intense precursor ions observed in the survey scan, which were subsequently put on an exclusion list for 5 min. Masses in this list were excluded with a mass accuracy window of ±10 ppm. The lock mass option was enabled and internal standard 1,1′,2,2′-tetramyristoyl cardiolipin was used as reference peak in negative mode at 619.4162 *m/z*. Prior to direct-infusion, lipid extracts were diluted 100-fold in LC-MS grade methanol and supplemented with 0.1% NH_4_OH or 0.1% formic acid plus 1 mM lithium acetate, for negative- or positive-mode ESI, respectively. Quantitation in pmol/g wet-weight was based on comparing precursor full-scan peak heights, with the internal standard amount set to 2 pmol.

## Results

### Construction of *Δcg2767/Δcg0049* Strain and Characterization of Its Growth Phenotype

*Cg2767* is the last gene of a predicted operon consisting of, from upstream to downstream, *cg2772*, *cg2770*, *cg2768*, and *cg2767*. *Cg2767* was only partially ablated by recombination due to an overlap of its downstream region with the upstream region of the predicted MarR-type regulator *cg2766*. The transcript of *cg0049* is predicted to be monocistronic, and the gene was completely deleted from the chromosome of the Δ*cg2767* mutant to obtain *C. glutamicum* ATCC13032 Δ*cg2767*/Δ*cg0049*. Phenotypic differences were assessed by strain cultivations at 30°C on minimal medium I (Supplementary Figure 1). The obtained growth curves for *C. glutamicum* ATCC13032, Δ*cg2767*, and Δ*cg2767*/Δ*cg0049* were virtually identical for all three strains. A further growth test on CgXII medium gave the same result (data not shown). Thus, it can be concluded that rhomboids do not affect growth of *C. glutamicum* under the tested conditions. Since this observation does not exclude the possibility of phenotypic differences on the molecular level, we opted for comparative proteome studies of the wild type and the double deletion mutant under various growth conditions (exponential phase, stationary phase, and heat stress) and further gathered transcriptome and lipidome data to complement our results.

Of note, introduction of chromosomal deletions always comes with the risk of causing unwanted secondary effects. In case of *cg2767*, there was the possibility of gene overlap with a putative regulator for downstream gene *cg2766*. For this reason, transcriptomes for Δ*cg2767/*Δ*cg0049* strain and WT were investigated for changes in expression of *cg2766*. There was minimal downregulation (factor −1.79) at 30°C and minimal upregulation (factor 1.61) at 40°C, thus expression of *cg2766* was not significantly affected by truncation of *cg2767*.

### Cg2767 Abundance Increases During Heat Stress

**FIGURE 1:**
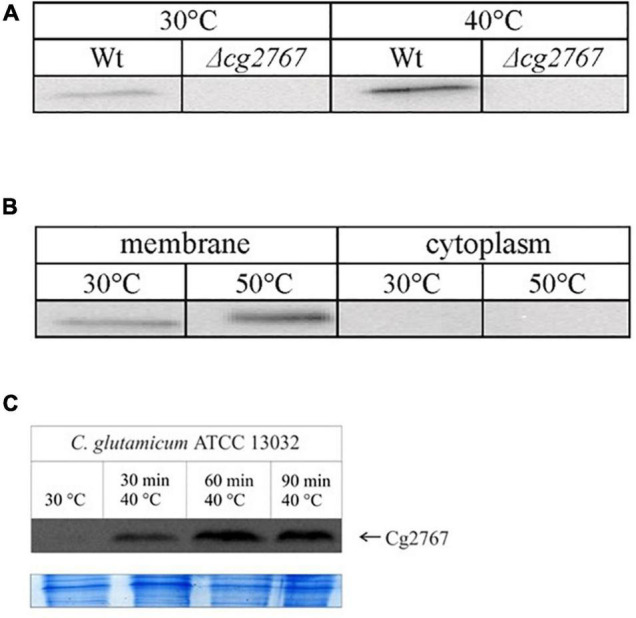
Western blots demonstrating increased abundance of Cg2767 upon heat stress. Membrane fractions of 25 μg protein were subjected to SDS-PAGE and Western blotting. (A) Detection of Cg2767 in the stationary growth phase at 30 and 40°C in Wt and Δ*cg2767* strain. (B) Detection of Cg2767 in the exponential growth phase at 30 and 50°C in membrane and cytoplasmic fraction of WT strain. (C) Time course analysis for Cg2767 at 40°C in the exponential growth phase and Coomassie-stained gel area for loading control; start point of sampling (30°C, *t* = 0 min) is indicated in Supplementary Figure 1. The protein signal was detected at a MW of 26 kDa exclusively in the Wt membrane fraction.

Increased rhomboid abundance should give a hint on its relevance under a given physiological condition. We failed to identify Cg0049 and Cg2767 with the described proteomics protocol most likely due to a combination of low protein amount and location of trypsin cleavage sites. Indeed, upon digestion with the proteases chymotrypsin or elastase (Fischer et al., 2006; Rietschel et al., 2009), we unequivocally identified Cg2767 in the membrane fractions (data not shown), and obtained low scoring peptide matches for Cg0049 – suggesting higher abundance of Cg2767 when cells are cultivated in minimal medium. As an alternative approach, differential synthesis of Cg2767 was probed with western blotting using a polyclonal antibody raised against the protein C-terminus. Presence of Cg2767 was confirmed with blotting under several tested conditions (cold shock, osmotic shock, different growth phases – data not shown) yet only for mild (40°C) and severe (50°C) heat stress protein amounts significantly differed from the standard growth temperature, both in the exponential and stationary growth phase. According to densitometry analysis of the *C. glutamicum* membrane fraction on western blots (Figure 1), mild or severe heat stress caused an about two-fold increase in the Cg2767 amount. Since this result suggests the participation of rhomboids in heat stress adaptation, this growth condition was included in the proteome analysis to reveal the physiological function of rhomboids in *C. glutamicum*.

### *Δcg2767/Δcg0049* Proteome Analysis, General Results

The absence of a growth phenotype suggested that physiological effects of rhomboid deletion may be more subtle, and in case of Cg2767 related to heat stress adaptation. Therefore, physiological differences between the WT and the double deletion strain, at ambient and elevated temperature (30 vs. 40°C) were investigated with quantitative shotgun proteomics after subcellular fractionation. By means of an isotope-labeled internal standard, about 80% of all identified proteins could be quantified. Supplementary Figure 2 gives an overview of proteins quantified with QuPE for each investigated condition and the corresponding cytoplasmic and membrane fraction. Each sample was measured as three biological replicates with the mean number of quantified proteins per sample being at least 850. Furthermore, mean number of quantified proteins was about 140 for the secretome.

Principal component analysis (PCA) analysis was done to verify the existence of a strain-dependent effect on proteome data variance and to address its contribution – the lower the component number, the more it characterizes the data distribution. Although there is no causal link between a PCA component and one of multiple experimental variables (strain, temperature, growth phase), in practice agreement between PCA component and experimental variable indicate cases, where such assumption is reasonable. In Supplementary Figure 3, component 2 is plotted against component 3 for the different subcellular fractions and growth conditions, whereas plots of the components 1 and 2 are depicted in the Supplementary Figure 4. The plots reveal that the components 2 and 3 separate the samples according to growth temperature and strain. Of note, in the exponential growth phase component 3 separates the different strains (WT vs. rhomboid deletion), while it is component 2 in the stationary growth phase. Stronger protein abundance changes (Supplementary Table 1) in the stationary compared to the exponential growth phase underpin these findings and suggest a more important role of rhomboids for stationary phase adaptation of *C. glutamicum*. For this reason, only proteome results for the stationary phase are presented in more detail, whereas they can be found in the supplement for the exponential phase.

### Transcriptomics of *Δcg2767/Δcg0049* in the Exponential Growth Phase at 30 and 40°C

Several genes related to iron and amino acid homeostasis changed their expression, and for these among other selected genes results will be presented here; the complete set of significantly changed gene expression can be found in Supplementary Table 2.

At 30°C, increased expression of the transcription factor *ripA* (*cg1120*) involved in iron homeostasis, and repression of a known target, nitrate reductase, was observed. Additionally, two iron-siderophore uptake systems, *cg0771* and *cg1418*, and a proteolytic subunit of Clp-protease (*clpP2, cg2644)* increased in expression. At 40°C, for one subunit of nitrate reductase (*cg1342*) more mRNA was detected. Uptake systems of ammonium or amino acids had lower expression: the putative secondary ammonium transporter *amtB* (*cg2261*), ABC-type putative amino acid transporter (*cg2340*), putative ABC-type peptide transporter (*cg0486*), and tryptophan permease *trpP* (*cg3357*). Two genes functioning in stress response, universal stress protein *UspA2 (cg1595*) and phosphate starvation-inducible protein *phoH2* (*cg2513*) displayed higher mRNA levels. Ferredoxin–NADP(+) reductase (*cg3119*) and putative ferredoxin reductase (*cg2999*) both increased in expression.

All in all, transcript levels were only slightly affected: the strongest upregulations/downregulations were detected for the hypothetical protein – containing START domain with putative polyketide synthase or lipid-binding function – *cg3124* (log_2_ −2.56) the transcriptional regulator ripA *cg1120* (log_2_ 2.05). Since deletion of the rhomboid genes only mildly affected gene expression, in the following result presentation focus is on proteome.

### *Δcg2767/Δcg0049* Proteome Dynamics in the Stationary Growth Phase at 30 and 40°C

Given the greater effect of rhomboids on the proteome in the stationary phase, selected proteins with different abundances in the WT and rhomboid deletion strain are presented below according to growth temperature and general protein function (COG category).

#### Energy Production and Conversion

Subunits of the succinate dehydrogenase complex (Cg0445, Cg0446, Cg0447) and menaquinol/cytochrome c-oxidase supercomplex (Cg2403, Cg2405, Cg2409, Cg2780) decreased in the MF at 30 and/or 40°C, whereas some subunits increased in the CF. Components of the pyruvate/2-oxoglutarate dehydrogenase complex (Cg1280, Cg2421) increased their amount in the MF, whilst Cg1280 decreased its amount in the CF 40°C. Increased amounts of pyruvate carboxylase (Cg0791) and phosphoenolpyruvate carboxylase (Cg1787) suggest replenishment of the TCA cycle with oxaloacetate in the deletion strain.

#### Cell Cycle Control, Cell Division, Chromosome Partitioning

Less dividing cells may be present in the deletion strain cultivated at 30°C, since predicted ATPase involved in cell division FtsE (Cg0914) and putative cell division initiation protein (Cg2275) were less abundant in the MF.

#### Amino Acid Transport and Metabolism

Several enzymes of amino acid biosynthesis pathways were more abundant in the MF or CF, for instance aspartokinase (Cg0306) at 40°C, aspartate-semialdehyde dehydrogenase (Cg0307) at 30°C, homoserine dehydrogenase (Cg1337) at 30 and 40°C, acetylglutamate kinase (Cg1582) in the CF and MF at 30°C and argininosuccinate synthase (Cg1586) in the MF at 30 and 40°C. Larger amounts of homocysteine methyltransferase (Cg1290), participating in methionine biosynthesis, were found in the MF at 30 and 40°C, but the opposite in the CF. PAPS reductase (Cg3115), involved in cysteine biosynthesis was less abundant in the MF at 40°C. In line with these findings, several other enzymes for amino acid biosynthesis increased their abundance in the CF in the rhomboid deletion strain: asparagine synthase at 30 and 40°C (Cg2410), glutamine synthetase (Cg2447) at 40°C, cysteine synthetase (Cg2833) at 40°C, tryptophan synthase beta chain (Cg3363) at 30°C. These results are corroborated by decreased abundance of relevant transporters: ArgK arginine/ornithine transport system ATPase (Cg1724), glutamate uptake system (Cg1236, Cg1239), and other amino acid/peptide transporters (Cg1281, Cg2937). Moreover, peptidases increased in amounts, such as aminopeptidase N (Cg2662) and aspartyl aminopeptidase (Cg1693) in the CF, whereas leucyl aminopeptidase (Cg2419) decreased in the MF and putative thiol precursor dipeptidase (Cg3201) in the CF, respectively.

Based on these findings, it can be hypothesized that the wild type can potentially better take up amino acids, whereas the rhomboid deletion strain requires their biosynthesis or liberation from proteins and peptides for sufficient amino acid supply.

#### Nucleotide Transport and Metabolism

Most likely the deletion strain synthesized more nucleotides at 40°C *de novo*, due to the increase of several biosynthesis enzymes for purine/pyrimidine and purine nucleosides, among them GMP synthase (Cg0703) in the MF at 30°C, phosphoribosylaminoimidazole synthetase PurM (Cg2856) in the CF at 40°C, phosphoribosylformylglycinamidine synthase PurL (Cg2862), CTP synthase (Cg1606) in the MF at 40°C, Uridylate kinase (Cg2218) in the MF at 30°C, and dihydroorotate dehydrogenase (Cg1713) in the CF at 40°C. Lower amounts of nucleoside diphosphate kinase (Cg2603) at 30°C and the nucleotide-salvage enzyme hypoxanthine-guanine phosphoribosyltransferase (Cg2985) at 30 and 40°C corroborate the aforementioned hypothesis.

#### Carbohydrate Transport and Metabolism

Some glycolytic enzymes changed their abundance: fructose-2,6-bisphosphatase (Cg0519), 3-phosphoglycerate kinase (Cg1790), glyceraldehyde-3-phosphate dehydrogenase (Cg1791), and pyruvate kinase (Cg2291) increased in the MF at 30°C, glucose-6-phosphate isomerase (Cg0973) decreased in the MF at 40°C, polyphosphate/ATP-dependent glucokinase (Cg2091) decreased in the MF at 30 and 40°C, glyceraldehyde-3-phosphate dehydrogenase (Cg1069) decreased in the CF at 30°C. Components of an ABC-type maltose uptake system (Cg2704, Cg2705) and ABC-type trehalose uptake system (Cg0832, Cg0834) were less abundant in the CF as well as MF at 30 and/or 40°C. Since 4-alpha-D-((1- > 4)-alpha-D-glucano)trehalose trehalohydrolase (Cg2333) was present at much higher amounts in the MF at 30°C, and 4-alpha-glucanotransferase (Cg2523) in the MF at 40°C, these results indicate remodeling of the peptidoglycan.

#### Coenzyme Transport and Metabolism

Strong increases of ketopantoate hydroxymethyltransferase (Cg1049) and putative phosphopantothenoylcysteine synthetase (1907) in the CF suggest elevated pantothenate biosynthesis.

#### Translation, Ribosomal Structure and Biogenesis

Many ribosomal subunits were significantly less abundant in the CF and MF at 30 and 40°C, the only exception was the ribosomal protein L6P/L9E (Cg0629) with higher abundance in the CF, but lower in the MF at 40°C. The amounts of several tRNA-synthetases changed in the rhomboid deletion strain – increases and decreases were found.

#### Transcription

Amounts of alpha, beta, and beta’ subunits of DNA-directed RNA (Cg0655, Cg0576, Cg0577) polymerase decreased in the CF and MF at 30 and/or 40°C, which hints at lower mRNA synthesis rates in the deletion strain. The regulator GntR (Cg1935) was much less abundant in the MF at 40°C. The regulators OxyR (Cg2109) and MalR (Cg3315) were less abundant in the CF at 40°C.

#### Posttranslational Modification, Protein Turnover, Chaperones

An overall larger amount of proteolytic enzymes was detected, such as ClpP2 (Cg2644) in the MF at 30 and 40°C, and periplasmic serine protease (Cg2043) in the CF at 40°C, while Co-chaperonin GroES (Cg0690) was less abundant in the CF at 30 and 40°C. Subunits of an ABC-type transport system involved in Fe-S cluster assembly (Cg1762 and Cg1763) were more abundant in the MF at 30 and 40°C. Thus, elevated proteolysis seems to occur in the deletion strain.

#### Inorganic Ion Transport and Metabolism

Apparently, ferric ion import mechanism differed in the two strains: ABC-type putative iron(III) dicitrate transporter (Cg0405) and ABC-type putative iron(III) dicitrate transporter (Cg2318) decreased at 30°C, however, ABC-type enterochelin transport system (Cg0924) was more abundant at 30°C. ABC-type cobalt transport system (Cg1228) and membrane protein TerC (Cg2157) were much less abundant at 40°C; concerning sulfur metabolism, sulfate adenylate transferase (Cg3114) was less abundant in the MF at 40°C, sulfite Reductase (Cg3118) more abundant in the CF, and sulfate permease (Cg1195) in the MF at 30°C. Amounts of periplasmic component of ABC-type phosphate transport system (Cg2846) strongly increased in the CF at 40°C.

#### Signal Transduction Mechanisms

Phosphate starvation response regulator PhoR (Cg2888) and a protein tyrosine phosphatase (Cg0415) were less abundant in the CF at 30°C. A predicted membrane GTPase involved in stress response in the CF and the universal stress protein UspA (Cg3255) in the MF were found in lower amounts at 40°C.

#### Intracellular Trafficking, Secretion, and Vesicular Transport

Preprotein translocase subunit SecA (Cg0868) and preprotein translocase subunit YajC (Cg1868) decreased their abundance in the MF at 30°C, suggesting overall decreased protein secretion in the deletion strain. Signal recognition particle GTPase (Cg2262) increased in the CF at 40°C.

#### Cell Wall/Membrane/Envelope Biogenesis

An enzyme apparently involved in regulation of cell wall biogenesis (Cg0418) was present at elevated level in the MF at 40°C. Three enzymes involved in peptidoglycan biosynthesis (Cg2368, Cg2373, Cg2374), UDP-galactopyranose mutase (Cg3196), and glycosyltransferase participating in cell wall biogenesis (Cg0438) were present in higher amounts in the CF at 40°C, whilst this was true for cyclopropane-fatty-acyl-phospholipid synthase (Cg0663) at 30°C. These findings are in line with the earlier assumed peptidoglycan remodeling.

#### Lipid Transport and Metabolism

Acetyl/propionyl-CoA carboxylase (Cg0802) and Acetyl-CoA carboxylase (Cg0812) decreased in the MF, whereas 3-oxoacyl-[acyl-carrier protein] reductase Fas-IB (Cg0957) increased in the MF, but decreased in the CF at 30 and 40°C, yet 3-oxoacyl-[acyl-carrier protein] reductase Fas-IA (Cg2743) increased in the CF at 40°C. Enoyl-CoA hydratase/carnitine racemase (Cg1049) was much more abundant in the CF at 30 and 40°C – altogether these data suggest decreased lipid biosynthesis and change in fatty acid composition. An enzyme involved in the deoxyxylulose pathway of isoprenoid biosynthesis (Cg2206) and deoxyxylulose-5-phosphate synthase (Cg2083) were detected at substantially increased amounts in the CF at 40°C, or in case of isopentenyl diphosphate isomerase (Cg2531) at 30°C.

### Comparative Shotgun Lipidomics of *Δcg2767/Δcg0049* in the Stationary Growth Phase at 30 and 40°C

**FIGURE 2:**
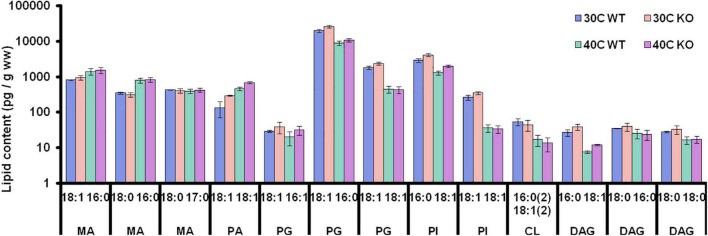
Lipidome of WT and rhomboid KO strain in the stationary phase. Lipidome alterations in the wild-type and Δcg2767/Δcg0049 *C. glutamicum* strain exposed to 30 and 40°C. Lipids were isolated using hot n-butanol extraction and spiked with defined amounts of synthetic lipid standards for semi-quantitative determination (in pg/g wet-weight), based on precursor peak intensities. Samples were analyzed with an LTQ Orbitrap mass spectrometer using high-resolution precursor scanning followed by HCD fragmentation. Interpretation of precursor and fragment spectra was performed using LipidXplorer, identifying 16 lipid species (MA, mycolic acid; PG, phosphatidylglycerol; CL, cardiolipin; PA, phosphatidic acid; PI, phosphatidylinositol; and DAG, diacylglycerol).

Given the detected abundance changes of enzymes involved in lipid, fatty acid, and peptidoglycan metabolism, rhomboids appear to affect cell envelope composition. To verify and substantiate this, shotgun lipidomics was employed for both *C. glutamicum* strains after cell harvest in the stationary phase. To this end, we determined and quantified various membrane constituents, such as mycolic acid, phosphatidylglycerol, cardiolipin, phosphatidic acid and phosphatidylinositol, containing predominantly C16 and C18 fatty acids (Figure 2). It is evident that both growth temperature and strain background influence *C. glutamicum*’s lipidome. As expected, when exposed to heat-stress *C. glutamicum* decreases phosphatidylglycerol levels, whilst simultaneously increasing mycolic acid content. With respect to the wild-type strain, phosphatidylglycerol, phosphatidic acid, diacylglycerol, and phosphatidylinositol were increased irrespective of cultivation temperature.

## Discussion

In most prokaryotes, the physiological function and the substrates of rhomboids are still ill-defined. In this first functional genomics analysis of corynebacterial rhomboid deletion strains, we pursued a multi-omics approach to uncover the involvement of the two rhomboid proteins Cg0049 and Cg2767 in heat stress and stationary phase adaptation of *C. glutamicum.* Even though under the tested conditions no obvious phenotypic differences could be observed, the obtained results demonstrate that rhomboid gene deletion affects a wide range of cellular functions on the transcriptome and proteome level in a growth stage and stress-specific manner. In the following, the physiological roles of rhomboids will be discussed with an emphasis on the involvement of Cg2767 in heat stress adaptation and lipid homeostasis. Moreover, the consequences of rhomboid gene deletion for *C. glutamicum* and concordances with other bacteria will be addressed.

### Consequences of Rhomboid Gene Deletion

**FIGURE 3:**
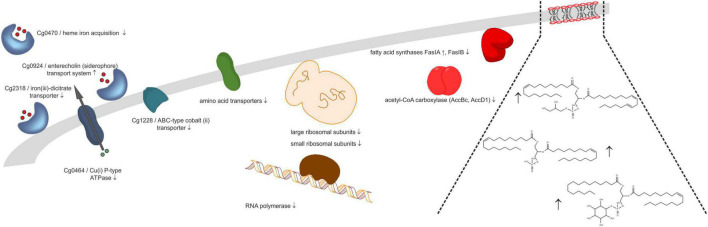
Rhomboids affect molecular composition of cell envelope. Proteins involved in ion homeostasis, amino acid transport, transcription and translation, and lipid metabolism, as well as plasma membrane lipids changed in abundance (up or down arrows, *Δcg2767/Δcg0049 C. glutamicum* vs. wild type ratio) upon deletion of the two *C. glutamicum* rhomboid genes.

Under the four tested conditions (30/40°C, exp./stat. phase), several physiological functions were commonly affected by rhomboid gene deletion, whereas other effects were unique to the respective condition. Regarding the number and extent of significantly changed protein abundances, the data demonstrate extensive involvement of rhomboids in stationary phase adaptation, and less importance for exponential growth, which agrees well with the observed indistinguishable growth behavior of wild type and rhomboid deletion strain before stationary phase. This observation is comparable to findings for *Mycobacterium smegmatis* (Kateete et al., 2012), where only biofilm formation but not growth in liquid culture was affected by rhomboid gene deletion. Figure 3 displays changes in protein abundance and possible consequences of rhomboid deletion for membrane-related processes. One common effect is changing, mostly decreasing, the relative amount of various ribosomal subunits already in the exponential, and even more the stationary phase. Because RNA polymerase decreased significantly under almost all conditions, too, a probable outcome could be decreased rates of RNA synthesis and protein synthesis in the rhomboid-deficient strain. We speculated that these proteome changes would render the strain more susceptible to antibiotics inhibiting protein synthesis, but could not detect different growth in agar plate diffusion assays with kanamycin (data not shown). Apparently, changes of proteins of transcription and translation machinery are not relevant for antibiotic susceptibility, or are compensated by unknown mechanisms. Another possibility is that *C. glutamicum* grown on agar plates might differ in the proteome from liquid culture, but this has not been investigated before. Another common consequence was an abundance change in cell wall-modifying enzymes. Again, we followed up this observation by antibiotic susceptibility tests, but no impaired growth was observed on agar plates incubated with penicillin or ampicillin (data not shown). Since expression of *cg0049* and *cg2767* is not affected upon exposure to ethambutol (Radmacher et al., 2005b), rhomboid expression levels seem to be irrelevant for the response and susceptibility to cell wall-targeting antibiotics.

### Putative Involvement of Cg2767 in Heat Stress Adaptation

In light of upregulated Cg2767 levels upon heat stress, it was reasonable to assume considerable proteome differences among both strains, yet this expectation could only be partially confirmed in our experiments: during exponential as well as stationary growth, more proteins demonstrated significantly altered abundance in the cytoplasmic fraction at 40°C compared to 30°C. However, such an effect was not obvious in the membrane compartment. Of note, protein abundance changes did not exhibit opposite trends at 30 and 40°C, i.e., no protein significantly increased in abundance at 30°C and significantly decreased at 40°C and *vice versa.*

Hence, it can be hypothesized that Cg2767 – perhaps together with Cg0049 – shapes the proteome at both temperatures in a similar way; yet at 40°C the effect is bigger. No results from other bacteria confirming the role of rhomboids in heat stress adaptation are available, but it has been found that expression of the plant rhomboid AtRBL10 increases during heat shock and 29% of its expression-correlated genes function in response to heat (Knopf and Adam, 2012), and its gene disruption affects lipid composition due to a potential role in a lipid synthesis complex assembly (Lavell et al., 2021). It remains to be verified if bacterial rhomboids exert related functions. It seems reasonable to assume that changes in membrane lipid composition due to heat stress affect activity profile of rhomboids given that lipids influence rhomboid conformation and PA might even pre-occupy the substrate binding site (Bondar, 2020).

### Putative Involvement of Rhomboids in Lipid Homeostasis

Shotgun lipidomic analyses of butanolic lipid extracts from *Δcg2767*/*Δcg0049* and WT *C. glutamicum* cells exposed to 30 and 40°C are discussed in relation to proteome data in the following section. *C. glutamicum* uses two different multisubunit carboxylase complexes for the supply of malonyl-CoA, and for the condensation of two fatty acids to mycolic acid, respectively. The former consists of the subunits AccbC (Cg0802) and AccD1 (Cg0812), the latter of AccbC (Cg0802), AccD2 (Cg0811), AccD3 (Cg3177), and AccE (Cg0810) (Gande et al., 2007). The malonyl-CoA is utilized for fatty acid synthesis with Fas-IB (Cg0957) or Fas-IA (Cg2743). Isolated Fas-IA synthesizes stearate and oleate, while Fas-IB synthesizes palmitate besides stearate (Radmacher et al., 2005a). In the exponential phase, two enzymes involved in mycolic acid biosynthesis increased in abundance. This could lead to increased amounts of mycolic acids in the cell wall envelope, and – with the additional decrease of peptidoglycan hydrolyzing enzyme Cg3424 at 30°C – make it less permeable for molecules.

In the stationary phase, decrease in Fas-IB at 30 and 40°C, and an increase in Fas-IA at 40°C were detected. Still the relative proportion of lipids remained largely unaffected with a relative increase in PA (18:1, 18:1) being the most prominent change. Perplexingly, Fas-IA is capable of producing unsaturated fatty acids in *C. glutamicum*; an additional desaturase is not required (Radmacher et al., 2005a). Since the desaturation mechanism of Fas-IA is completely unknown, we can merely record the increased level of unsaturated fatty acids even upon heat stress in the absence of rhomboids.

Glycerol phosphate acyltransferase (GPAT) upregulation, which subsequently transfers newly synthesized acyl chains from acyl-CoA to glycerol-3-phosphate to form acyl-glycerol 3-phosphate, are clear indicators of increased lipid biosynthesis. Indeed, as shown in Supplementary File 3, PA as key intermediate of phospholipid biosynthesis, was approx. 2-fold higher in butanolic lipid extracts from *Δcg2767*/*Δcg0049*, as compared to WT (*p* = 0.06). Concomitantly, downstream PG levels were 30% higher observed in the double deletion strain (as listed in Supplementary File 3). With respect to mycolic acid (MA) content, which is reported to increase extracellularly with culture age during the late stationary phase (Jang et al., 1997), in both wild type as well as the double deletion strain higher MA levels were observed upon heat stress, suggesting regulation of MA content by rhomboids in addition to SigD (Taniguchi et al., 2017). Apparently, *C. glutamicum* heat-stress response differs from other metabolic stress conditions, such as amino acid overproduction, where MAs have been shown to decrease, resulting in increased permeability of *C. glutamicum*’s cell surface layer (Hashimoto et al., 2006). Given the larger amounts of AccBC and AccD1 alongside the lower amount of crotonase (Cg1049) in the mutant, not only a divergent fatty acid synthesis profile, but also lower rates of *de novo* fatty acid synthesis, and higher rates of fatty acid oxidation can be expected.

**FIGURE 4:**
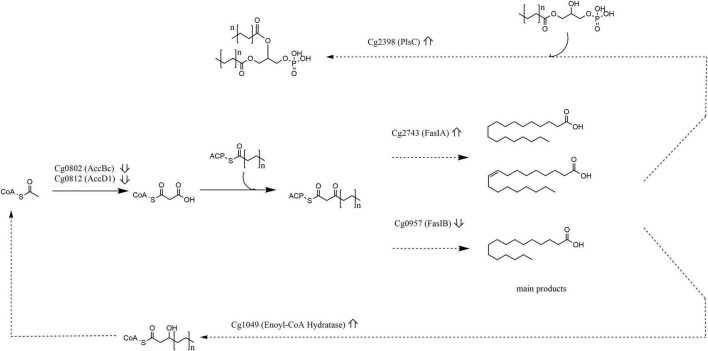
Lipid metabolism is affected upon rhomboid gene deletion. Excerpts of lipid metabolism showing protein abundance changes (ratio Δcg2767/Δcg0049 *C. glutamicum* vs. wild type up or down arrow). Enzymes involved in acyl-CoA synthesis (AccBc, AccD1), fatty acid synthesis (FasI, FasIB), fatty acid degradation (Cg1409), and phospholipid biosynthesis (PlsC) were affected.

Taken together, ablation of rhomboid function in *C. glutamicum* results in distinct metabolic changes at the level of phospholipid production (Figure 4). As such, our finding shed new light on current endeavors to use *C. glutamicum* as lipid production platform (Takeno et al., 2013) and on pathogenicity of relatives (Burkovski, 2018), in that key enzymes of the lipid biosynthesis pathway (Figure 4) differing in abundance between the WT and *Δcg2767*/*Δcg0049* strain are direct targets or indirectly regulated by rhomboids. This might contribute to the high lipid diversity of corynebacteria (Klatt et al., 2018; Wang et al., 2020).

In conclusion, our data attributes rhomboids a regulatory function for cell wall and membrane lipid synthesis with their deletion causing higher membrane fluidity during heat stress in the stationary phase, and probably a more rigid cell wall during exponential phase.

### Function in Respiratory Chain Protein Quality Control

In *S. sonnei* rhomboid function in membrane protein complex quality control was demonstrated (Liu et al., 2020). These and previous findings for mitochondria led the authors speculate about evolutionary conserved function of rhomboids in quality control of respiratory complexes. Our results for the succinate dehydrogenase would agree with this hypothesis: impaired complex assembly or removal of orphan subunits might have occurred upon rhomboid deletion during heat stress, since one subunit (Cg0447) decreased in the MF, yet increased in the CF. Moreover, observed decrease of cytochrome-bc1-aa3 supercomplex subunits in MF reveals rhomboids affect directly or indirectly other *C. glutamicum* respiratory chain members.

### Rhomboid Function in Pathogenic Relatives

The very little knowledge about rhomboid function in related coryne- and mycobacteria is mostly from expression and genome analysis. According to a comprehensive genome and expression analysis, majority of mycobacteria, just like *C. glutamicum*, have two rhomboid genes that are expressed when grown in liquid culture (Kateete et al., 2010). Evidence for membrane rhomboid localization (Målen et al., 2010) and a non-essential function for *in vitro* growth (Griffin et al., 2011) was obtained for *M. tuberculosis* rhomboid Rv0110 (Målen et al., 2010). Likewise, its ortholog Rv1337 is non-essential for *in vitro* growth in rich media, yet is required for survival in primary murine macrophages (Rengarajan et al., 2005). Single and double deletions of the rhomboid encoding genes *msmeg_5036* (ortholog of *cg0049*) and *msmeg_4904* (ortholog of *cg2767*) were constructed in the related *M. smegmatis* (Kateete et al., 2012). Deletion of *msmeg_4904* rendered the bacterium more susceptible to the DNA gyrase inhibitors Ciprofloxacin and Novobiocin, and decreased biofilm formation. Interestingly, strains carrying the deletion of *msmeg_5036* or the double deletion were less susceptible to antibiotics. From the scarce amount of existing data, it is hard to say to which extend functions and targets of rhomboids are conserved among *C. glutamicum* and its relatives. Phenotypes reported to date for *M. tuberculosis* and *M. smegmatis* are specific for their intracellular and biofilm lifestyle that differs from *C. glutamicum*.

## Conclusion

This study revealed that rhomboids are linked to a diverse set of cellular functions and modulate the composition of the transcriptome, proteome, and lipidome in a growth state- and temperature-dependent manner. These results suggest the participation of rhomboids in a larger regulatory network that involves metal homeostasis, amino acid homeostasis, protein synthesis, and fatty acid synthesis and lipid composition. Future works are needed to untangle these regulations with identification of rhomboid substrates being the first priority. The obtained *C. glutamicum* ATCC13032 Δ*cg2767*/Δ*cg0049* provides an ideal chassis for the plasmid-based expression of inactivated and/or affinity-tagged rhomboids to validate substrate candidates from the current study and might even reveal function of rhomboids in larger membrane protein complexes, as reported for *B. subtilis* (Began et al., 2020). Pursuing such studies also with *M. tuberculosis* and *M. smegmatis* would shed light on unique and shared rhomboid substrates in related bacteria.

## Data Availability Statement

The datasets presented in this study can be found in online repositories. The names of the repository/repositories and accession number(s) can be found below: https://www.ebi.ac.uk/arrayexpress/, E-MTAB-2558, http://qupe.cebitec.uni-bielefeld.de, qupe.rhomboide.

## Author Contributions

AP: conceptualization of study. AP, AL, and SA: proteome data analysis. AL: molecular biology, cultivation, sample preparation, and analysis. TN and VW: transcriptomics. FV: lipidomics. AL and AP: writing. AP and VW: reviewing and editing. All authors contributed to the article and approved the submitted version.

## Conflict of Interest

The authors declare that the research was conducted in the absence of any commercial or financial relationships that could be construed as a potential conflict of interest.

## Publisher’s Note

All claims expressed in this article are solely those of the authors and do not necessarily represent those of their affiliated organizations, or those of the publisher, the editors and the reviewers. Any product that may be evaluated in this article, or claim that may be made by its manufacturer, is not guaranteed or endorsed by the publisher.

## Footnotes

1
http://www.massmatrix.net

## Supplementary Material

Supplementary File 1 Table of significantly changing protein abundances.

Supplementary File 2 Table of significantly changing mRNA abundances.

Supplementary File 3 Lipidome of *C. glutamicum*.

Supplementary File 4 Growth curve of *C. glutamicum* strains, additional PCA results, and physiological impacts of rhomboid gene deletion.

Supplementary File 5 Description of proteomics results for exponential growth phase.
