# Supplementary material for: Functional Genomics Uncovers Pleiotropic Role of Rhomboids in Corynebacterium glutamicum
Source: Front Microbiol. 2022 Feb 21;13:771968. doi: 10.3389/fmicb.2022.771968 (PMC8899591; doi:10.3389/fmicb.2022.771968)
Supplement: Supplementary File 1 — Table of significantly changing protein abundances. [file Table_1.DOCX]

## *Δcg2767/Δcg0049* proteome dynamics during exponential growth at 30 °C and 40 °C

In the following sections, the observed proteome differences will be presented according to growth condition and general protein function (COG category). If trends, i.e. an increase or decrease, are identical and significant on the proteome and transcriptome level, protein IDs are printed in bold to indicate and confirm transcriptional regulation as underlying reason for observed protein abundance changes.

*Energy production and conversion:* Overall, the membrane fraction was most affected. Here, subunits of the pyruvate dehydrogenase complex (**Cg0441**, **Cg0790**, **Cg2421**), channelling pyruvate into the TCA cycle, and the citrate synthase (Cg0949) increased their abundance at 30 °C and 40 °C. Concerning protein levels of the respiratory chain, one subunit of succinate dehyrogenase (Cg0446) increased in the CF at 30 °C, two subunits of ATP synthase (**Cg1364**, Cg1367) decreased in the CF or increased in MF at 30 °C, cytochrome c (Cg2405) decreased in the secretome at 40 °C, and a subunit of cytochrome c oxidase (Cg2409) increased in the CF at 40 °C. According to these and other protein abundance changes, it appears that energy metabolism and perhaps also localization of few proteins differs between deletion strain and WT, in particular at 40 °C.

*Amino acid transport and metabolism:* Levels of aspartate semialdehyde dehydrogenase (Cg0307), acetolactate synthase (Cg1436), acetylglutamate kinase (Cg1582), arginine/ornithine transport system ATPase (Cg1724), cysteine synthase (**Cg2833**) were higher in the MF at 30 °C, or additionally at 40 °C for 4-amino-4-deoxychorismate lyase (Cg2418). The permease component of an ABC-type peptide transport system (Cg2677) was less abundant in the MF at 30 °C. These data suggest increased synthesis of several amino acids, but less peptide import in the deletion strain.

*Nucleotide transport and metabolism:* Increased uracil phosphorylation may occur in the deletion strain, since uracil phosphoribosyltransferase (Cg0786) was more abundant in the CF, and uridylate kinase (Cg2218) in the MF at 30 °C. Phosphoribosylcarboxyaminoimidazole (NCAIR) mutase (Cg0820) and aspartate carbamoyltransferase (**Cg1816**), were more abundant, inosine-uridine nucleoside N-ribohydrolase (Cg2168) was less abundant in the CF at 40 °C.

*Carbohydrate transport and metabolism:* Many proteins were less abundant in the rhomboid deletion strain and indicate reduced carbohydrate consumption: ABC-type trehalose permease (Cg0832) in the MF at 30 °C, glucose-6-phosphate dehydrogenase (Cg1779) and 3-phosphoglycerate kinase (Cg1790) in the CF at 30 °C, Fructose-1-phosphate kinase (Cg2119) in the CF at 40 °C.

*Coenzyme transport and metabolism:* Rhomboid deletion could influence heme biosynthesis due to more glutamate-1-semialdehyde aminotransferase (Cg0518) in the MF, and less uroporphyrinogen-III decarboxylase (Cg0516) in the CF. Furthermore, CoA biosynthesis (phosphopantetheine adenylyltransferase, Cg1501), thiamine biosynthesis (hydroxyethylthiazole kinase, Cg1655), and NAD biosynthesis (quinolinate synthase, **Cg1216**) were affected.

*Lipid transport and metabolism:* Acetyl-CoA carboxylase AccD3 (**Cg3177**) increased its abundance in the CF at 40 °C and 1-acyl-sn-glycerol-3-phosphate acyltransferase (Cg2398) and acyl-CoA synthetase FadD5 (Cg0480) increased their abundance in the MF at 30 °C, which alludes to differences in lipid composition between the two strains.

*Translation, ribosomal structure and biogenesis:* Some ribosomal subunits were significantly less abundant in the CF and MF at 30 °C, while for 40 °C no obvious trend was observed. At 40 °C, more tRNA and rRNA cytosine-C5-methylase (Cg1802) in the MF, less ribosome-binding factor A (Cg2175) in the CF were present. Aspartyl-tRNA synthetase (Cg1841) was less abundant in the CF and translation initiation factor 2 (Cg2176) more abundant in the MF at 30 °C.

*Transcription:* The amount of DNA-directed RNA polymerase (Cg0577) in the CF was less at 40 °C, even though its transcript level significantly increased. The MarR family regulator **Cg2766** was more abundant in the CF at 40 °C, but expression of its target genes (according to (31)) was not significantly affected. The LuxR family master regulator of carbon metabolism RamA (Cg2831) and DNA or RNA helicase of superfamily II (Cg2097) were more abundant in the MF at 30 °C.

*Replication, recombination and repair:* Topoisomerase IA (Cg0373) and helicase of DNA excision repair complex (Cg1550) in the MF, NAD-dependent DNA ligase (Cg1401) in the CF were all detected at elevated levels at 40 °C. Together with decreased amounts of RNA polymerase, the results suggest that during heat stress, DNA damage and decreased mRNA synthesis occurs in the deletion strain.

*Cell wall/membrane/envelope biogenesis:* The observed abundance changes support the idea of differences in peptidoglycan structure between the two strains: an enzyme apparently involved in regulation of cell wall biogenesis (Cg0418) at 30 °C increased in abundance in the MF, whereas cell division septal protein (**Cg2367**) decreased at 40 °C. In addition N-acetylmuramoyl-L-alanine amidase (Cg3424) decreased at 30 °C, and nucleoside-diphosphate-sugar epimerase (Cg3375) at 40 °C, whilst at 40 °C another nucleoside-diphosphate-sugar epimerase (Cg1740) increased in abundance.

*Posttranslational modification, protein turnover, chaperones:* A thioredoxin domain-containing protein (Cg1375) was present in lesser amounts at 40 °C in the CF, yet its gene expression was higher. In the MF, ATPase subunit of an ABC-type transport system involved in Fe-S cluster assembly (Cg1762) was more abundant at 30 °C, and stomatin/prohibitin homologous membrane protease (**Cg3138**) less abundant at 40 °C.

*Inorganic ion transport and metabolism:* In the MF, the periplasmic component of an ABC-type Fe3+-hydroxamate transport system (**Cg0771**) increased its abundance at 30 °C. In the CF, periplasmic component of ABC-type enterochelin transport system (Cg0924) was present in larger amounts at 40 °C, whilst ferritin-like protein (Cg2782) was present in lesser amounts at 40 °C despite increased mRNA levels. Taken together, the deletion strain seems to exhibit increased uptake, yet decreased storage, of iron at 40 °C.

*General function prediction und function unknown:* The amounts of many proteins with unknown function changed upon deletion of rhomboid genes, but these changes hardly provide further insight into the physiological function of rhomboids. Worth mentioning is the quite strong decrease in protein amount of a tryptophan-specific permease (**Cg3357**) in the MF at 40 °C.

*Signal transduction mechanisms:* Carbon starvation protein (**Cg0756**) and the phosphate starvation response regulator PhoR (Cg2888) were less abundant in the MF at 40 °C, although *phoR* expression increased – thus starvations decreased or their response was hampered in the deletion strain. Response regulator of the OmpR family with unknown function (Cg0996) was more abundant in the CF at 30 °C.

*Intracellular trafficking, secretion, and vesicular transport:* In the MF, preprotein translocase subunit SecY (Cg0647) at 30 °C, and preprotein translocase subunit YidC at 40 °C decreased in amount. Even though this could be interpreted as impaired protein secretion, the overall accordance of secretome data can only fit to this hypothesis if the translocation impairment rather equally affects all secreted proteins.
