# Supplementary figures and images for: Functional Genomics Uncovers Pleiotropic Role of Rhomboids in Corynebacterium glutamicum
Source: Front Microbiol. 2022 Feb 21;13:771968. doi: 10.3389/fmicb.2022.771968 (PMC8899591; doi:10.3389/fmicb.2022.771968)

## Slide 1
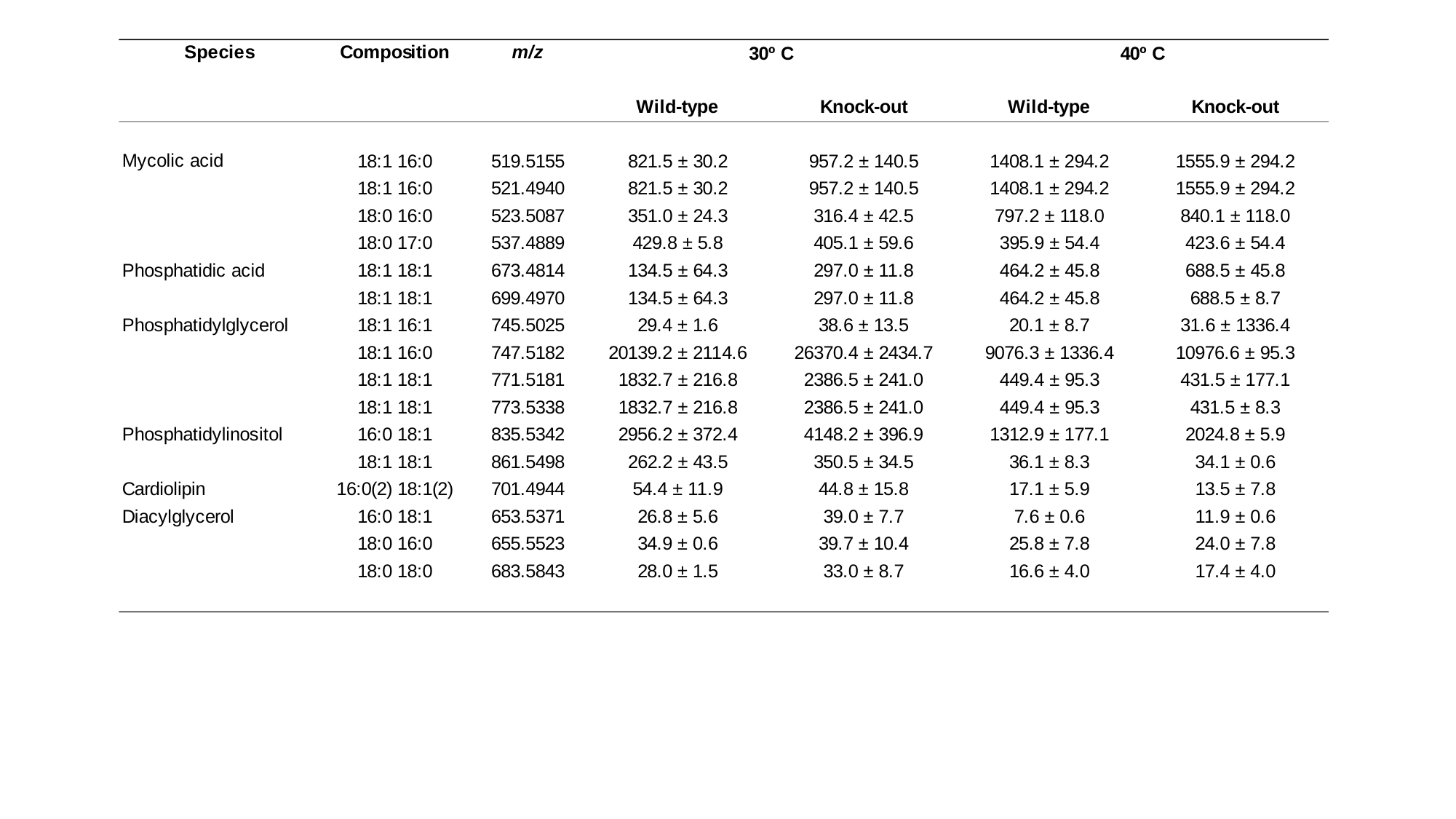

Supplement: Supplementary File 4 — Growth curve of C. glutamicum strains, additional PCA results, and physiological impacts of rhomboid gene deletion. [file Presentation_1.PPTX]
