# Supplementary material for: Functional Genomics Uncovers Pleiotropic Role of Rhomboids in Corynebacterium glutamicum
Source: Front Microbiol. 2022 Feb 21;13:771968. doi: 10.3389/fmicb.2022.771968 (PMC8899591; doi:10.3389/fmicb.2022.771968)
Supplement: Supplementary file 5 [file Presentation_2.PPTX]

## Slide 1
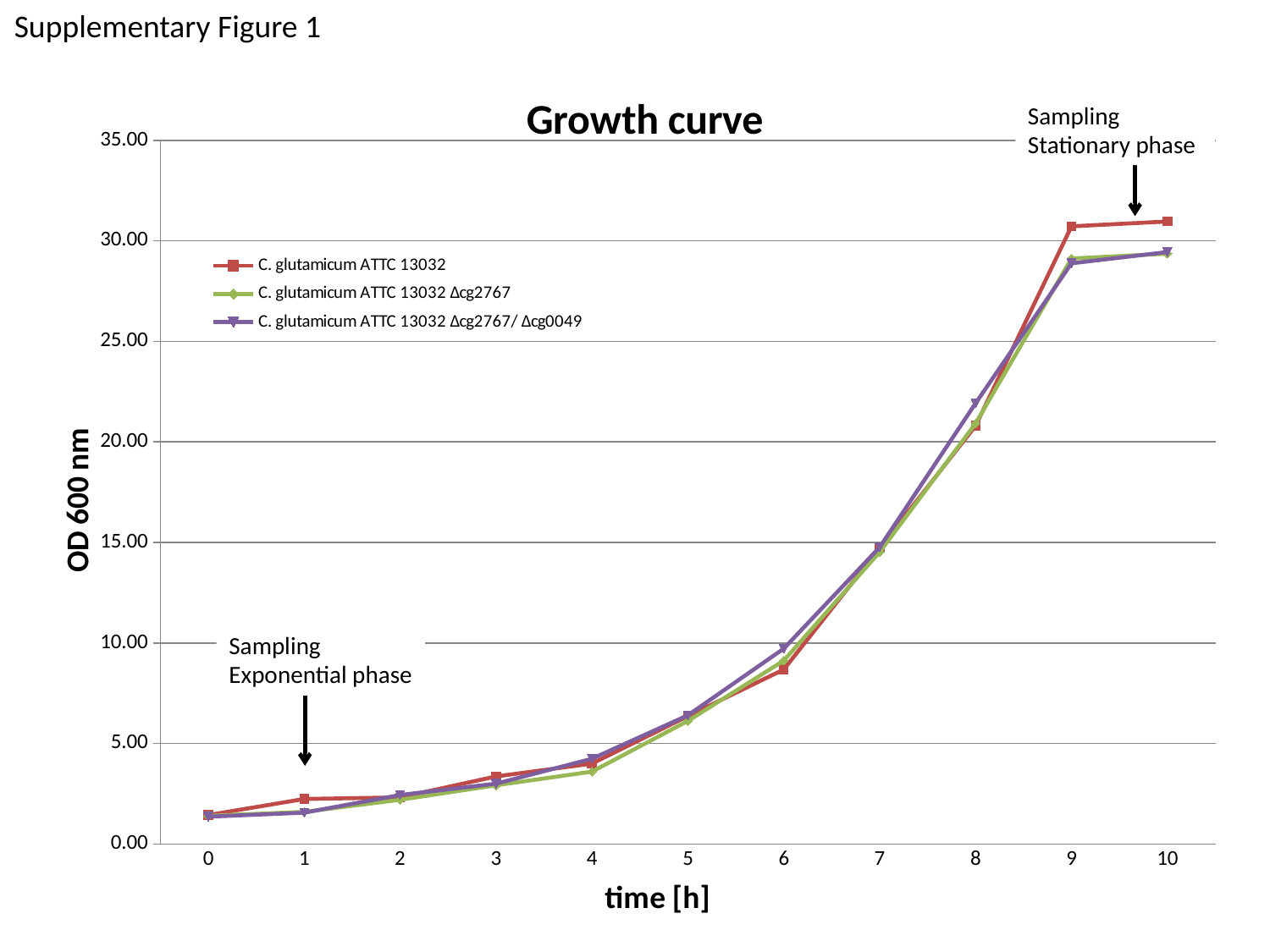

Supplementary Figure 1
### Chart: Growth curve
| Category | C. glutamicum ATTC 13032 | C. glutamicum ATTC 13032 Δcg2767 | C. glutamicum ATTC 13032 Δcg2767/ Δcg0049 |
|---|---|---|---|
| 0 | 1.44 | 1.4000000000000001 | 1.36 |
| 1 | 2.24 | 1.6 | 1.56 |
| 2 | 2.3200000000000003 | 2.2 | 2.44 |
| 3 | 3.3600000000000003 | 2.92 | 3.0 |
| 4 | 4.0 | 3.5999999999999996 | 4.24 |
| 5 | 6.36 | 6.12 | 6.4 |
| 6 | 8.68 | 9.120000000000001 | 9.719999999999999 |
| 7 | 14.719999999999999 | 14.52 | 14.76 |
| 8 | 20.8 | 20.92 | 21.92 |
| 9 | 30.72 | 29.119999999999997 | 28.88 |
| 10 | 30.96 | 29.36 | 29.439999999999998 |Sampling
Stationary phase
Sampling
Exponential phase

## Slide 2
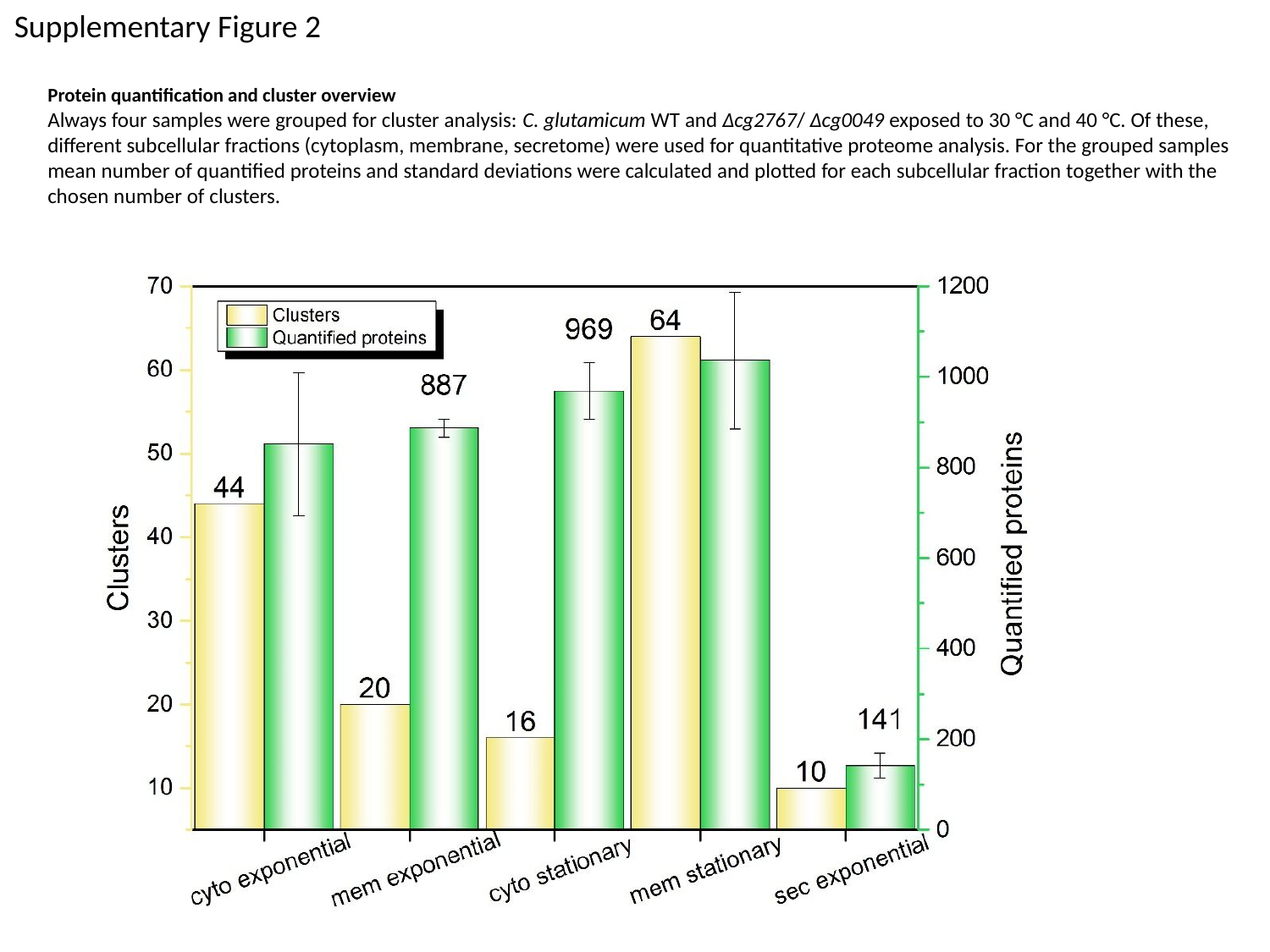

Supplementary Figure 2
Protein quantification and cluster overview
Always four samples were grouped for cluster analysis: C. glutamicum WT and ∆cg2767/ ∆cg0049 exposed to 30 °C and 40 °C. Of these, different subcellular fractions (cytoplasm, membrane, secretome) were used for quantitative proteome analysis. For the grouped samples mean number of quantified proteins and standard deviations were calculated and plotted for each subcellular fraction together with the chosen number of clusters.

## Slide 3
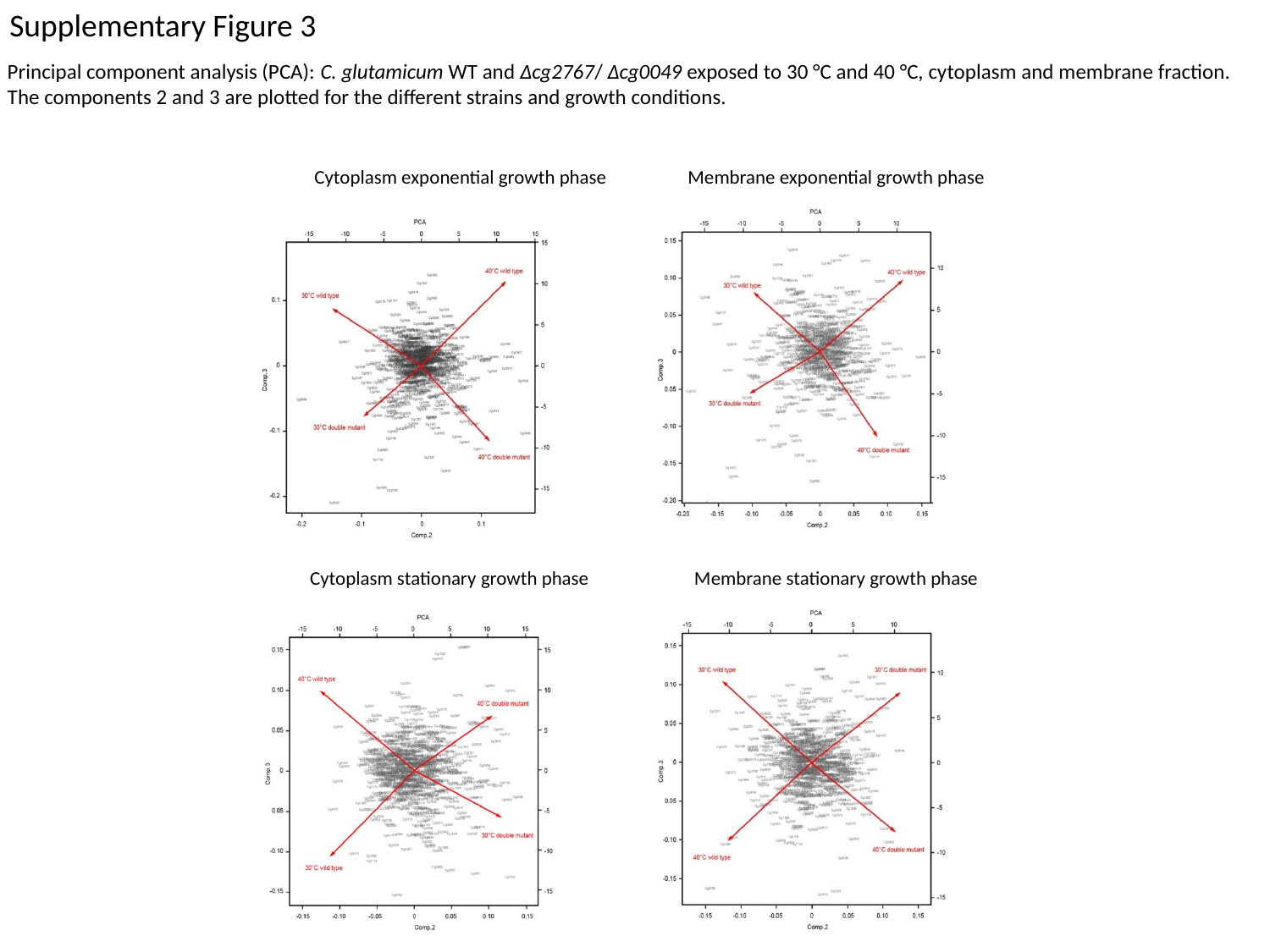

Supplementary Figure 3
Principal component analysis (PCA): C. glutamicum WT and ∆cg2767/ ∆cg0049 exposed to 30 °C and 40 °C, cytoplasm and membrane fraction.
The components 2 and 3 are plotted for the different strains and growth conditions.
Cytoplasm exponential growth phase
Membrane exponential growth phase
Cytoplasm stationary growth phase
Membrane stationary growth phase

## Slide 4
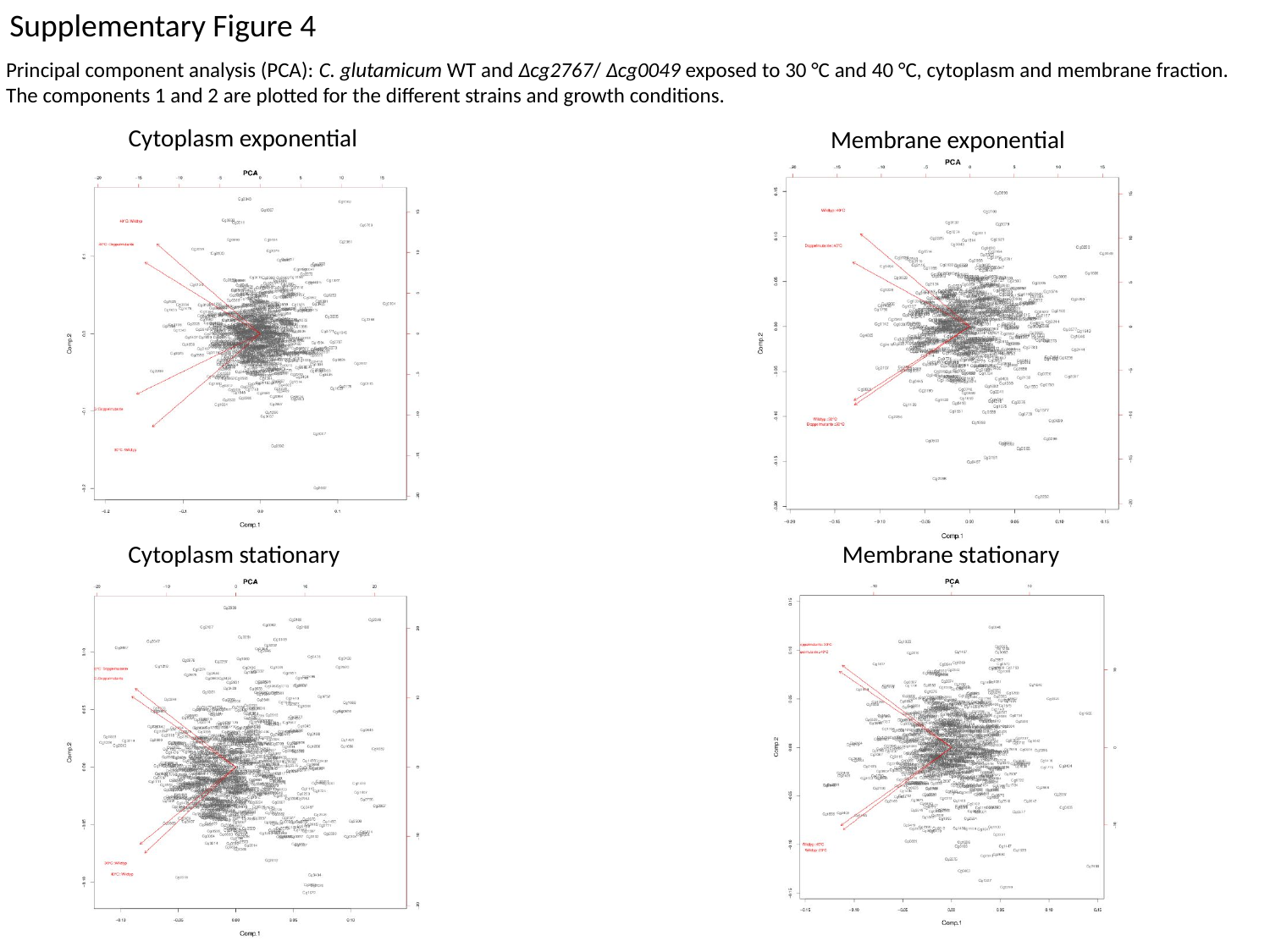

Supplementary Figure 4
Principal component analysis (PCA): C. glutamicum WT and ∆cg2767/ ∆cg0049 exposed to 30 °C and 40 °C, cytoplasm and membrane fraction. The components 1 and 2 are plotted for the different strains and growth conditions.
Cytoplasm exponential
Membrane exponential
Cytoplasm stationary
Membrane stationary

## Slide 5
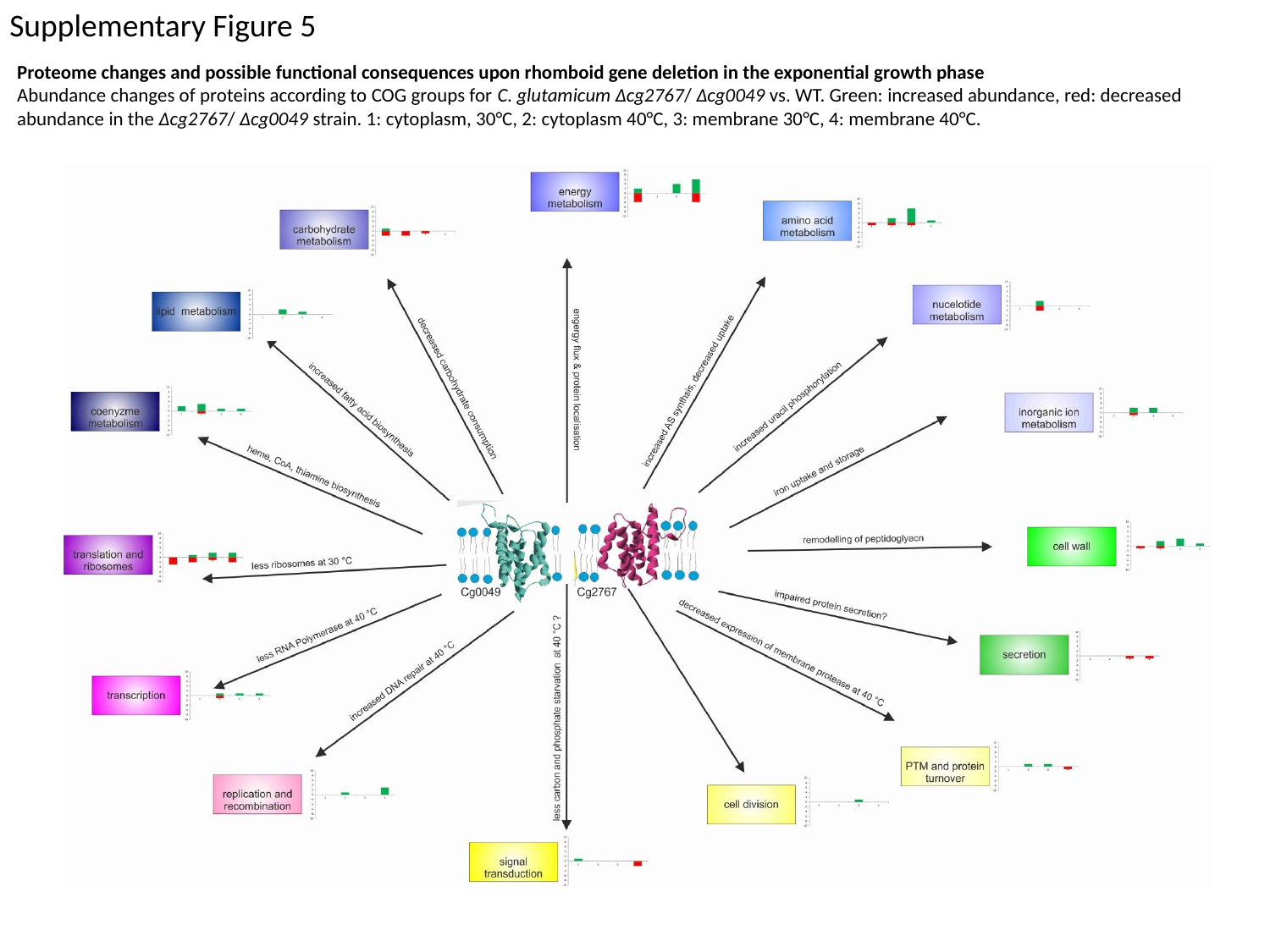

Supplementary Figure 5
Proteome changes and possible functional consequences upon rhomboid gene deletion in the exponential growth phase
Abundance changes of proteins according to COG groups for C. glutamicum ∆cg2767/ ∆cg0049 vs. WT. Green: increased abundance, red: decreased abundance in the ∆cg2767/ ∆cg0049 strain. 1: cytoplasm, 30°C, 2: cytoplasm 40°C, 3: membrane 30°C, 4: membrane 40°C.

## Slide 6
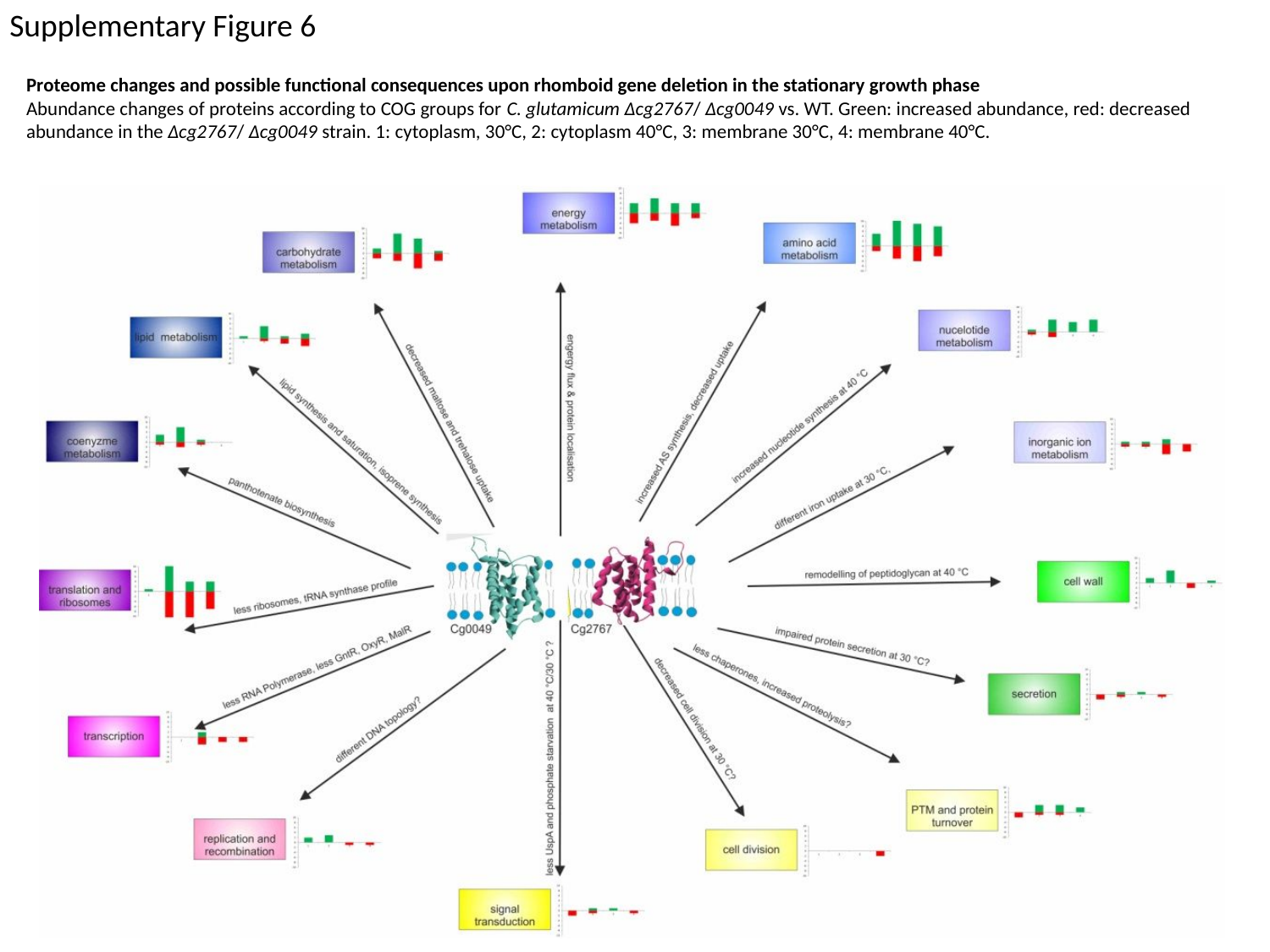

Supplementary Figure 6
Proteome changes and possible functional consequences upon rhomboid gene deletion in the stationary growth phase
Abundance changes of proteins according to COG groups for C. glutamicum ∆cg2767/ ∆cg0049 vs. WT. Green: increased abundance, red: decreased abundance in the ∆cg2767/ ∆cg0049 strain. 1: cytoplasm, 30°C, 2: cytoplasm 40°C, 3: membrane 30°C, 4: membrane 40°C.
